# Weakening Investigation of Reservoir Rock by Coupled Uniaxial Compression, Computed Tomography and Digital Image Correlation Methods: A Case Study

**DOI:** 10.3390/s21020344

**Published:** 2021-01-06

**Authors:** Peiwu Shen, Huiming Tang, Bocheng Zhang, Yibing Ning, Xuexue Su, Sixuan Sun

**Affiliations:** 1 Department of Engineering Geology and Geotechnical Engineering, Faculty of Engineering, China University of Geosciences, Wuhan 430074, China; pwshen@cug.edu.cn (P.S.); zhangbocheng@cug.edu.cn (B.Z.); yeebingning@cug.edu.cn (Y.N.); suxuexue@cug.edu.cn (X.S.); 20151000308@cug.edu.cn (S.S.); 2 School of Engineering, University of British Columbia, Kelowna, BC V1V 1V7, Canada; 3 Three Gorges Research Center for Geohazards, Ministry of Education, China University of Geosciences, Wuhan 430074, China

**Keywords:** reservoir rock, cyclic wetting and drying, CT, DIC, strength variation, structure variation

## Abstract

Cyclic wetting and drying treatment is commonly used to accelerate the weakening process of reservoir rock. The weakening is reflected in strength variation and structure variation, while the latter receives less attention. Based on a series of cyclic wetting and drying tests, this study tentatively applied the uniaxial compressive test, computed tomography (CT) test and digital image correlation (DIC) test to investigate the weakening of slate in a reservoir area. Test results show that the weakening is mainly reflected in the reduction of compressive strength, followed by the decrease of ability to resist cracking and elastic deformation. The weakening seems more likely to be caused by structure variation rather than composition change. Two failure modes, e.g., splitting and splitting-tension, are concluded based on the crack paths: the splitting failure mode occurs in the highly weathered samples and the splitting-tension failure mode appears in the low-weathered samples. The transition zones of deformation are inside samples. The nephogram maps quantify the continuous deformation and correspond to the aforementioned structure variation process. This study offers comprehensive methods to the weakening investigation of slate in reservoir area and may provide qualitative reference in the stability evaluation of related slate rock slope.

## 1. Introduction

In reservoir area, the fluctuating reservoir water level is one typical cyclic wetting and drying to the rock of reservoir bank slope, which expedites the physical weathering process and further induces the reservoir geohazards (e.g., landslide, collapse and debris flow). Most of the reservoir geohazards appear after impounding [1,2,3], and thus, the investigation on the weakening of rock exposed to cyclic wetting and drying has received much concern [4,5,6]. The weakening of rock resulted from cyclic wetting and drying can be reflected in two aspects: strength variation and structure variation. Currently, the strength variation in rock can be measured by various mechanical tests [7,8,9,10,11], e.g., the uniaxial compressive test, triaxial compressive test and direct shear test. Considering that most natural rocks are in the stress state of compression, the compressive tests are more commonly used. However, the structure variation in rock is rarely tested. For this consideration, two non-destructive and non-contact methods including the computed tomography (CT) and digital image correlation (DIC) are utilized in this study, to investigate the internal and external structure variations of weakened reservoir rock.

The CT method is applied to obtain the reconstruction image of an object [12,13,14]. In the scanning, an X-ray beam is used to scan a certain thickness of object, the penetrating X-ray is then received by a detector and is further converted into visible light and electrical signal so that one computer can recognize and process. The CT method was first used in medical diagnosis [12,15,16,17,18]. With the perfection of theory and operation, the CT method was introduced into the field of rock mechanics, which benefits the development of rock mechanics [19,20,21,22,23]. Currently, the application of the CT method in rock mechanics focuses on the visualization of rock damage, rock composition, rock stress state and rock test process, etc. [19,21,22,24,25]. The DIC method is developed to measure the full-field displacement of objects [26,27,28,29,30]. In the measurement, the point of the region of interest (ROI) between the reference image and current image is tracked, so that the displacement of this point and then the full field can be determined. Since the DIC method has a wide range of applications, some scholars applied the method to rock mechanics, and the current researches on the combination of the DIC method and rock mechanics concentrate on the deformation of rocks under various test conditions [31,32,33,34,35].

The application of CT and DIC methods contributes to the development of rock mechanics. In general, the CT method is suitable for internal structure investigation, while the DIC method is fit for external structure investigation. However, the application of the methods is often independent, and the comprehensive investigation of natural rock based on coupled mechanical treatment, CT method and DIC method is lacking, let alone the reservoir rock subject to physical weathering.

**Figure 1:**
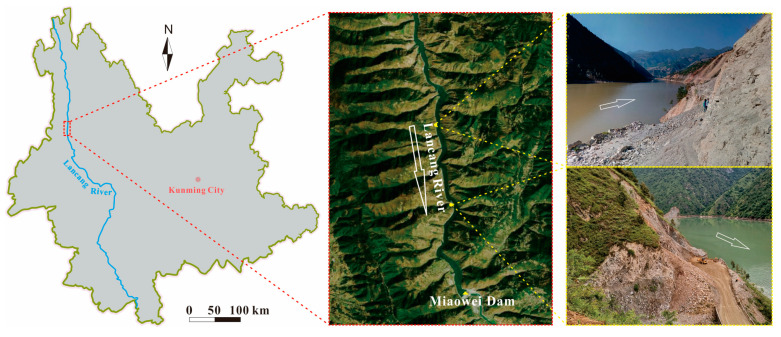
Location of Miaowei reservoir area in Yunnan province and two landslide sites.

**Figure 2:**
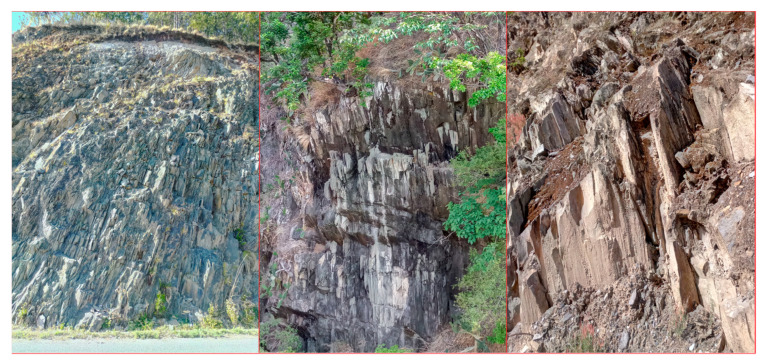
Exposed rocks of reservoir bank slopes in Miaowei reservoir area.

**Figure 3:**
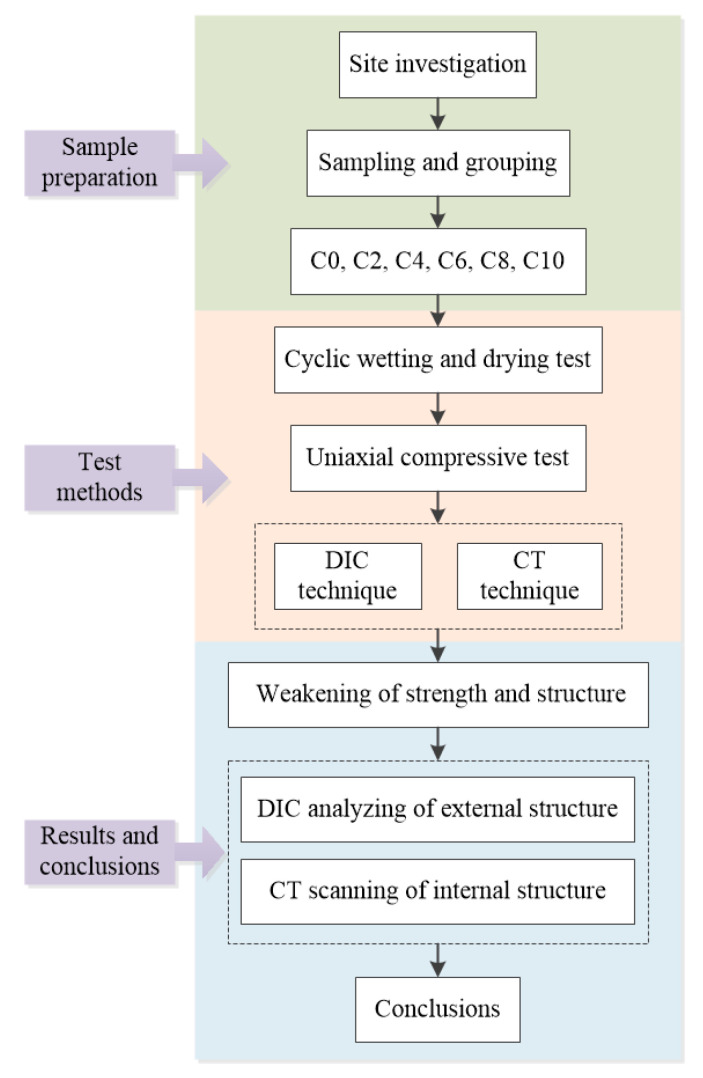
Flow chart of this study.

The Miaowei hydropower station in Yunnan province, southwestern China, operated in 2018. With the impounding of reservoir, some reservoir bank slopes have slid, which threatens the people and buildings nearby (Figure 1). Field investigation shows that most of the exposed rocks are slate and are steeply inclined with various weathering levels (Figure 2). This study investigated the weakening characteristics of slate in the Miaowei reservoir area using the coupled uniaxial compressive test, CT test and DIC test. As shown in Figure 3, the cyclic wetting and drying test was first conducted to make the samples in different levels of weathering; then, the uniaxial compressive test was carried out to learn the strength variation of samples, and the CT test and DIC test were executed to study the internal and external structure variation of samples; finally, one comprehensive understanding of the weakening characteristics of slate in the Miaowei reservoir area was investigated.

## 2. Sample Preparation and Test Methods

### 2.1. Sample Preparation

**Figure 4:**
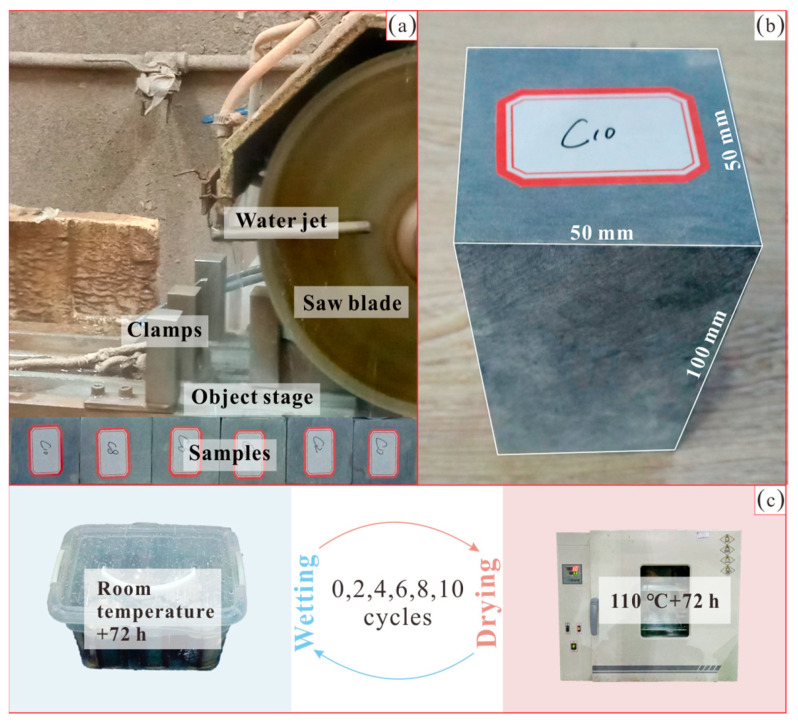
Sample preparation by cutting (**a**), sample size (**b**) and cyclic wetting and drying test (**c**).

The samples tested in this study are slate from the Miaowei reservoir area, with a natural density of 2.74 g/cm^3^ and the mineral composition of sericite, chlorite and quartz. In order to maintain the natural state, the fresh slate rock blocks after field sampling were sealed by plastic wrap and then boxed by foam board for transportation. As shown in Figure 4a,b, the rock blocks were finally cut into cube samples with the size of 50 mm × 50 mm × 100 mm (length × width × height) to fulfill laboratory test requirements, the samples were resealed and six identical samples named C0, C2, C4, C6, C8 and C10 were prepared in this study.

### 2.2. Test Methods

The tests including cyclic wetting and drying, uniaxial compression, DIC and CT were orderly conducted to investigate the weakening of samples. Although these test methods have been applied to rock mechanics, it should be noted that the cyclic wetting and drying test can speed up the weathering process of samples, but it cannot fully meet the weathering conditions of natural rocks. The uniaxial compression test is convenient to carry out and provides visual conditions for the real-time deformation observation of samples, however, most natural rocks are always in triaxial stress state. The CT test can be conducted to observe the internal deformation of samples, but cannot provide real-time observation data, while the DIC test can record the continuous deformation of samples in real time, but cannot monitor the inside deformation of samples. Therefore, these test methods need to be combined to make up for the shortcomings. These test methods are detailed in the following three parts.

#### 2.2.1. Cyclic Wetting and Drying Test

The cyclic wetting and drying test was conducted to accelerate the weathering process of samples so that significant weakening occurred. The samples named C0, C2, C4, C6, C8 and C10 were designed to respectively experience 0, 2, 4, 6, 8 and 10 cycles of wetting and drying, for the sake of possessing stepped levels of weakening. As shown in Figure 4c, the test includes two parts: (1) wetting and (2) drying. For the former, the samples were unpacked and then placed in containers with distilled water fully submerged, the wetting is set to room temperature and lasts 72 h without disturbance in each cycle and the water was replaced once per cycle. For the latter, the samples after wetting were moved to an oven with the temperature of 110 °C and the drying lasts 72 h without disturbance, the samples after each drying were air-cooled to room temperature in the oven. The wetting and drying were duplicated to designed cycles (0, 2, 4, 6, 8 and 10 cycles) so as to obtain six samples possessing stepped levels of weakening. The related controlling parameters of the cyclic wetting and drying test in this study are summarized in Table 1.

#### 2.2.2. Uniaxial Compressive Test

**Figure 5:**
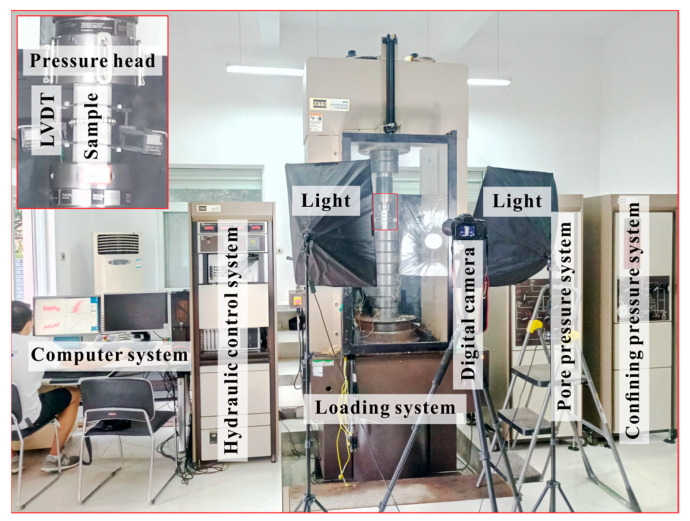
Apparatus for uniaxial compressive test.

**Figure 6:**
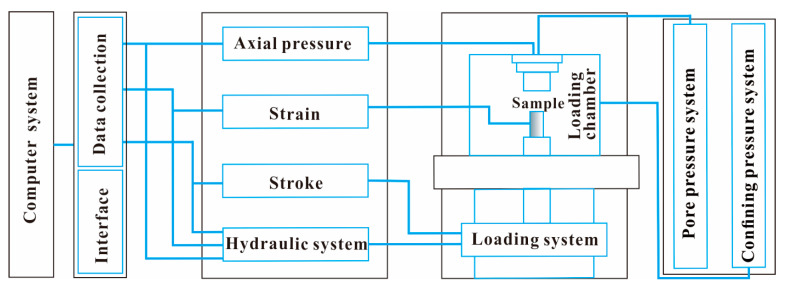
Structure diagram of Mechanics Testing System.

**Figure 7:**
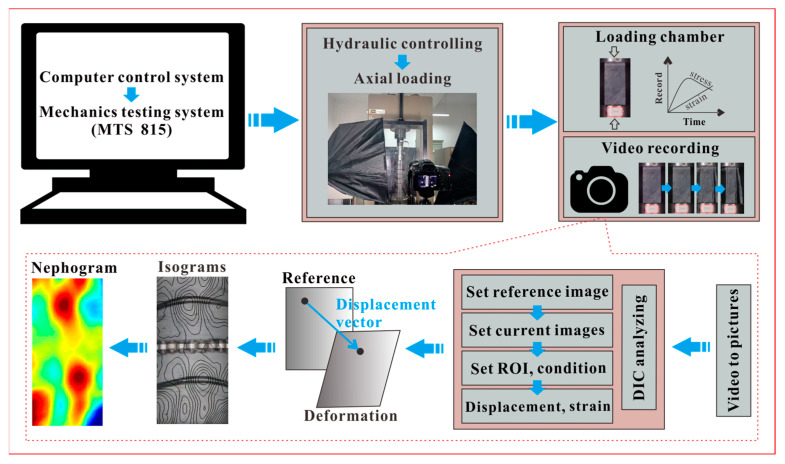
Digital image correlation (DIC) test and schematic diagram of test data processing.

The uniaxial compressive test was conducted to explore the strength variation of weakened samples, which also provides a reference for the later structure variation analysis. The samples were tested on the Mechanics Testing System 815 produced by MTS Systems Corporation (Eden Prairie, MN, USA). As shown in Figure 5, the Mechanics Testing System 815 is supported by five subsystems, e.g., the computer system, hydraulic system, loading system, pore pressure system and confining pressure system, and the related system structure diagram is shown in Figure 6. In this study, the computer system, hydraulic system and loading system were activated to complete the uniaxial compressive test, the test was computer-controlled and the data was automatically recorded and processed (Figure 7). In the test, the laboratory temperature was about 25 °C and the loading was displacement-controlled. The travel rate of the pressure head when contacting the sample was set to 0.002 mm/s so that the deformation of samples was easier to be caught by a digital camera. Other parameters of the operation are default values and more controlling parameters of the Mechanics Testing System 815 are displayed in Table 2. It is noted that the cracking stress, uniaxial compressive strength and elastic modulus were tested to analyze the strength variation. The cracking stress corresponds to the stress when crack initiates in samples, the uniaxial compressive strength is the maximum strength of samples under compression and the elastic modulus is the ability of samples to resist elastic deformation.

#### 2.2.3. CT Test

**Figure 8:**
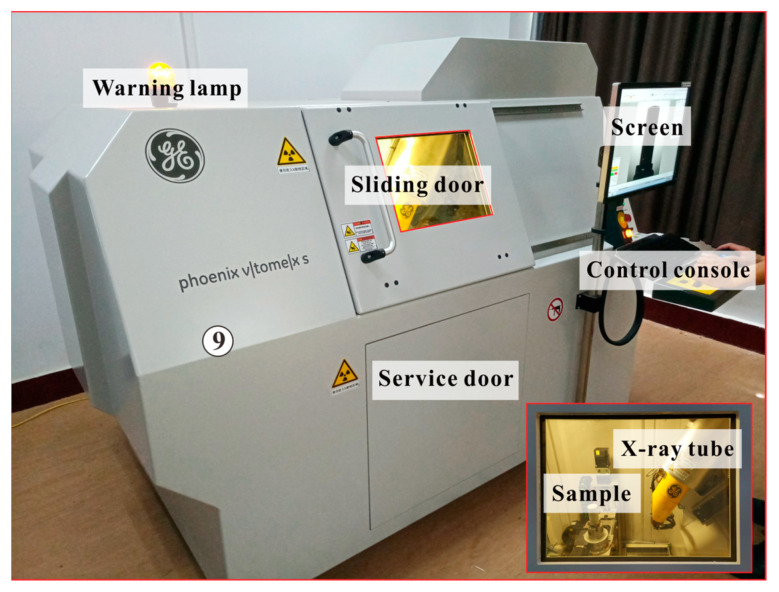
Apparatus for computed tomography (CT) test.

**Figure 9:**
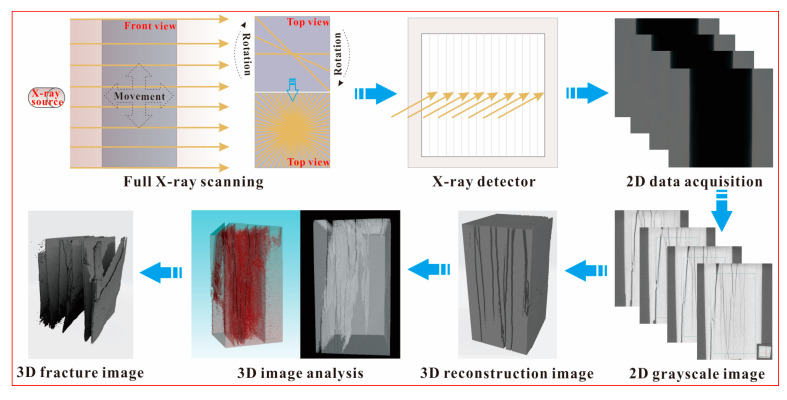
Schematic diagram of CT processing.

The CT test was conducted to investigate the internal structure variation of weakened samples. The samples were tested on the X-ray Inspection System v|tome|x s 240 (Figure 8) produced by GE Sensing and Inspection Technologies GmbH, Wunstorf, Germany. The system is computer-controlled and supported by four sets of software, e.g., the xs|control, datos|x acquisition, datos|x reconstruction and VGSTUDIO MAX; among which, the first three are built in the system and the last one is developed by Volume Graphics GmbH, Heidelberg, Germany. In the test, the xs|control was used to monitor and control the X-ray tube to scan, the datos|x acquisition was utilized to collect the two-dimensional (2D) CT image series (sectional projections) from the scanning, the datos|x reconstruction was applied to reconstruct the three-dimensional (3D) volumes of samples from the sectional projections and the VGSTUDIO MAX was developed for visualization and post-processing based on the reconstructed 3D volumes of samples. The samples can be scanned and reconstructed by the system since the X-ray displays dissimilar penetration ability to solid phase and gas phase. In general, the solid phase indicates the rock matrix of samples and the gas phase represents the crack (damage) of samples. The solid phase and the gas phase were combined to obtain the distribution of damage, so that further investigation on the internal structure variation was conducted (Figure 9). In this study, the laboratory temperature is 20 °C, and the voltage and current of the X-ray tube are set to 120 kV and 90 μA, respectively. Other parameters of the operation are default values and more controlling parameters of the system are displayed in Table 3.

#### 2.2.4. DIC Test

The DIC test was conducted to investigate the external structure variation of weakened samples, which is completely different from the uniaxial compressive test since the DIC test makes the deformation process reappeared and visible. As shown in Figure 5, the compression process of specimens was recorded by a digital camera (24 frames per second and 1920 × 1080 pixels per frame), which is the basis for further DIC analysis. The basic principle of DIC analysis is to track the displacement of target points (pixels) between the reference image (without deformation) and current images (with deformation) (Figure 7). Specifically, the video recording the full deformation process of one sample was first converted to a series of digital pictures; then, the image showing the sample without deformation was set as the reference image, while the images displaying the sample experiencing continuous deformation were used as the current images, the number of current images can be determined by the required deformation interval of one sample. After that, the correlation criteria was applied to catch the displacement vectors of target points in the ROI; finally, the continuous deformation process of one sample can be quantified by regional deformation, which was intuitively represented by the isograms and nephogram maps. The DIC test was conducted based on an open-source DIC algorithm [28] and two representative samples with an obvious difference in weakening level were tested.

## 3. Results

### 3.1. Strength Variation

**Figure 10:**
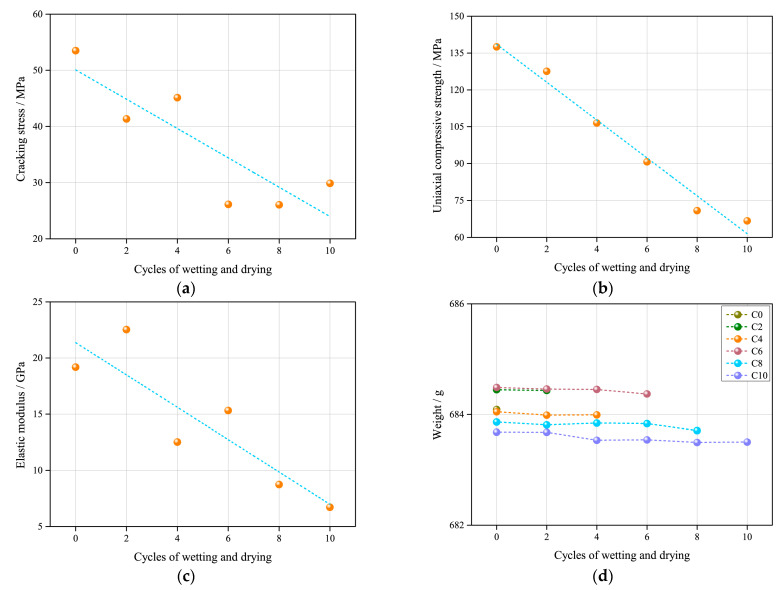
Correlations of cracking stress (**a**), uniaxial compressive strength (**b**), elastic modulus (**c**) and weight (**d**) versus cycles of wetting and drying.

Figure 10a–c shows the cracking stress, uniaxial compressive strength, elastic modulus and weight versus the cycles of wetting and drying. In this study, it is noted that the cracking stress, uniaxial compressive strength and elastic modulus show an overall decreasing trend with the growth of cycles of wetting and drying. However, the cracking stress and elastic modulus have fluctuant variation trends, the uniaxial compressive strength shows a more monotonous variation trend; specifically, the cracking stress seems to vary between 53.5 and 26.1 MPa, the uniaxial compressive strength likely changes between 66.8 and 137.5 MPa and the elastic modulus seems to alter between 6.7 and 22.5 MPa. The difference indicates that the cyclic wetting and drying has the greatest impact on the compressive strength of samples, while in the long term, the cyclic wetting and drying also weakens the ability of samples to resist cracking and elastic deformation. Figure 10d also demonstrates that cyclic wetting and drying has almost no effect on the weight of samples since all the samples basically remain unchanged in terms of weight. The result further reveals that the weakening of the samples experiencing cyclic wetting and drying is not mainly reflected in the erosion of composition, but may be the change of structure, which can be further explored from the CT test and DIC test.

### 3.2. Structure Variation

**Figure 11:**
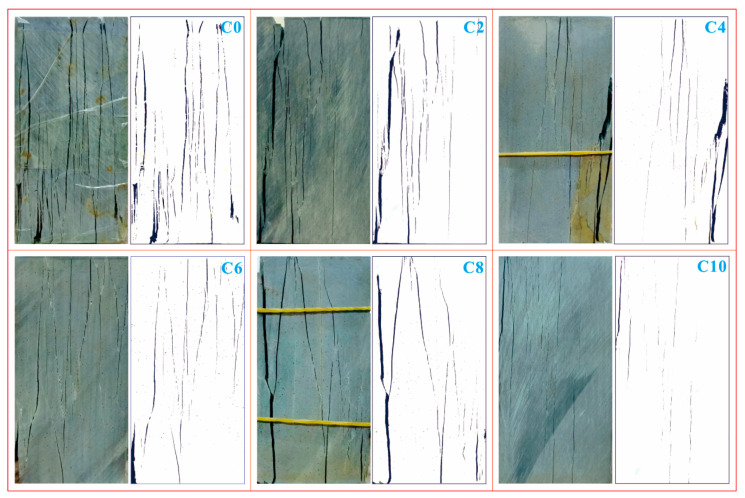
Samples after uniaxial compressive test and related crack sketches.

**Figure 12:**
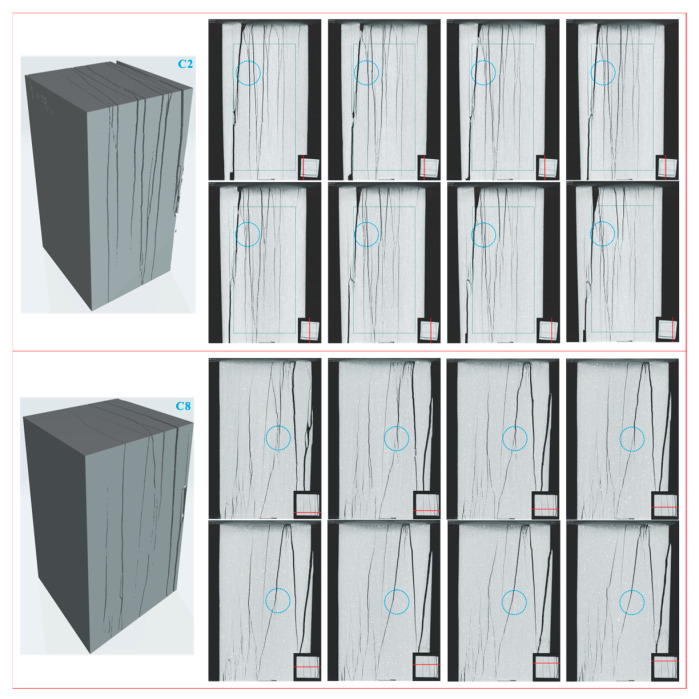
Spatial distribution of cracks inside samples, red line at bottom right corner indicates longitudinal section’s position in top view.

The structure variation is embodied in the exterior damage and interior damage of samples, which is jointly analyzed by the DIC test and CT test. The samples after the uniaxial compressive test provide the most intuitive material for the analysis of exterior damage. Figure 11 shows the samples and the corresponding damage sketches after the uniaxial compressive test. It is obtained that the samples with lower weathering levels tend to display more complex failure modes. In particular, the samples experiencing fewer cycles of wetting and drying possess more obvious cracks on the surface and the cracks are more widespread; usually, the cracks get coalescent on the surface and cause greater damage. With the increasing of cycles of wetting and drying, the cracks on the surface of samples are less coalescent and the related damage levels decrease. These cracks not only appear on the surface of samples, but also inside the samples, which can be seen from Figure 12. Figure 12 shows the continuous longitudinal sections of two representative samples after two and eight cycles of wetting and drying respectively, and the red line at the bottom right corner indicates the position of each longitudinal section in the top view. It is observed that most of the cracks inside samples are coalescent. The sample after fewer cycles of wetting and drying contains more cracks that are interlaced, compared with the sample experiencing more cycles of wetting and drying. This phenomenon is because as the cycles of wetting and drying increase, the initial micro-cracks gradually propagate along foliation planes, which finally causes pre-damage in samples before the uniaxial compressive test. Once the samples are subjected to external compression, the developed micro-cracks tend to quickly grow into macro-cracks along foliation planes, which eventually leads to the rapid destruction of samples; however, for the samples experiencing fewer cycles of wetting and drying, most of the micro-cracks do not go through the pre-damage along foliation planes; thus, the micro-cracks have the opportunity to fully propagate and interlace under compression, which results in the wide distribution of cracks and the high damage levels of samples.

It is concluded that the samples behave both by splitting failure mode and splitting-tension failure mode. For the splitting failure mode, the crack path basically follows foliation planes, the cracks are far apart and generally do not interfere with each other. This failure mode appears in the samples that have experienced more cycles of wetting and drying. For the splitting-tension failure mode, the crack path is partly along foliation planes and partly between foliation planes. This is because the crack spacing is small and the splitting along foliation planes easily transforms into tension between foliation planes. This failure mode arises in the samples that have undergone fewer cycles of wetting and drying.

**Figure 13:**
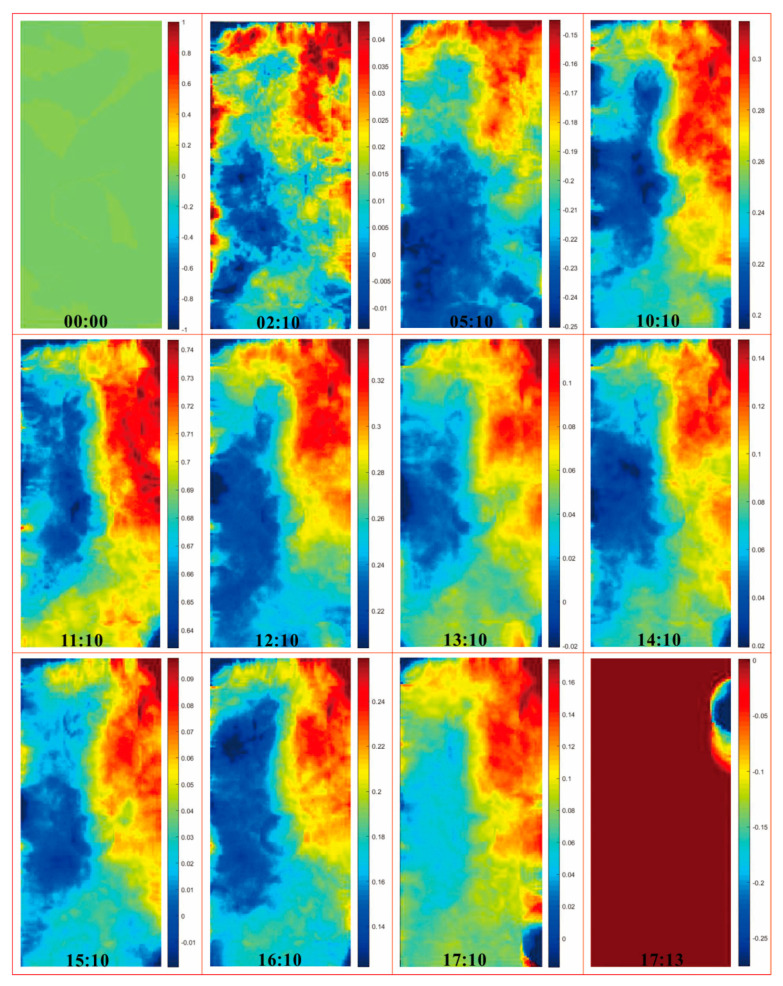
Nephogram maps of axial displacement of C2.

**Figure 14:**
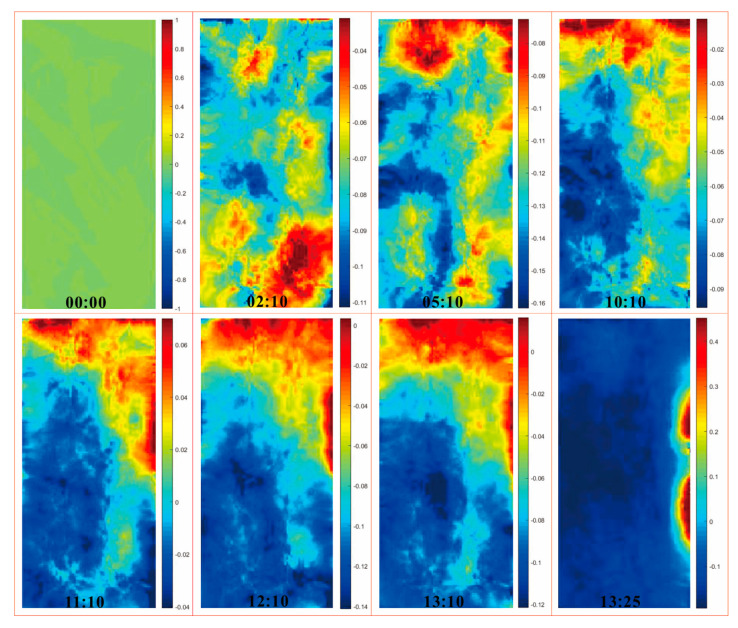
Nephogram maps of axial displacement of C8.

The above results from direct observation and the CT test can be further verified in the DIC test. Figure 13 and Figure 14 show the nephogram maps of axial displacement of the samples, the same as in Figure 12. The compressive direction defaults to be positive and the units are in millimeters. As the test time shows in the lower middle of each frame, the sample experiencing more cycles of wetting and drying takes less test time to reach destruction compared with the sample undergoing fewer cycles, which corresponds to the aforementioned analysis. From the perspective of axial displacement, it is clear that the obvious deformation areas are relatively scattered at the beginning—this is because the positions of micro-cracks are random. As compression continues, the obvious deformation areas gradually get connected due to the coalescence of cracks and the deformation is basically along foliation planes. After that, the deformation areas of a sample that has experienced more cycles expand along foliation planes until the sample is entirely split and destroyed, then the deformation areas gradually disappear, and the test is over. The deformation of a sample that has undergone fewer cycles of wetting and drying continues, and the deformation areas are further connected; at this time, the coalescence areas tend to stagger foliation planes until the split-tension failure runs through the entire sample, then the deformation areas disappear, and the sample is destroyed. The results from the DIC test correspond to the results from the CT test, which together reveal the structure variation of samples.

The results from the CT test can be used not only to verify the macroscopic failure modes of samples, but also to analyze the evolution modes of local cracks inside samples. As shown by the cracks in circles of Figure 12, the same crack will show different evolution modes during the propagating process inside samples; specifically, a single crack may generate multiple branch cracks inside one sample, and multiple branch cracks may eventually gather into one crack. The turning point of this change generally appears inside samples rather than on the surface. This phenomenon indicates that the stress state inside one sample is not the same, and usually makes the failure mode of a sample more complicated. For the single-crack region, the propagation of a crack will cause the sample to show a pure deformation mode (e.g., splitting mode), while for the multi-crack region, the propagation of a crack will cause the sample to show a combined deformation mode (e.g., splitting-tension mode), the transition zone of deformation is generally inside the sample and is difficult to be observed in conventional mechanical tests. The evolution phenomenon of a crack in a sample has a good corresponding relationship with the engineering geological condition; for example, due to the influence of in-situ stress, the deformation of high and steep slate rock slopes often manifest as combined deformation modes including the combined splitting and toppling deformation. Identifying the deformation zones of rock can better study its engineering properties; however, the transition zone of deformation is also not easy to be observed on site, which makes the current related researches mainly based on generalized models. The analytical results in this study help to understand the deformation process, failure mode and deformation transition zone of slate after cyclic wetting and drying, which may provide reference to the stability evaluation of slate rock slope in a reservoir area.

## 4. Conclusions

This study tentatively used the coupled uniaxial compressive test, CT test and DIC test to investigate the weakening characteristics of reservoir rock. The test methods and apparatuses were first introduced in detail, then the strength and structure variations based on the tests were studied. Several valuable conclusions were obtained and summarized, as follows.

The physical weathering affects the strength of samples under compression, which was recorded in the uniaxial compressive test. The cracking stress, uniaxial compressive strength and elastic modulus show decreasing variation trends with the increasing cycles of wetting and drying. The weakening degree of compressive strength is higher than that of the cracking resistance and elastic deformation resistance. The physical weathering is more inclined to weaken the structure rather than erode the composition.

The physical weathering varies the crack paths of samples under compression, which was visualized in the CT test and the DIC test. Both splitting failure mode and splitting-tension failure mode occurred in the samples, the former makes the crack path basically follow foliation planes, and the latter causes the crack path to be partly along foliation planes and partly between foliation planes. The samples with lower weathering levels possess more widespread cracks on the surface and inside, and the cracks are more interlaced than that of the samples with higher weathering levels. The transition zones of deformation are generally inside samples, which indicates the multiple stress states in samples under compression. The quantified deformation process represented by nephogram maps helps prove the aforementioned analytical results.

The cyclic wetting and drying test, uniaxial compressive test, CT test and DIC test conducted in this study provide new insight into the mechanism investigation of weakening of reservoir rock. The method can also be applied to other reservoir rocks for further qualitative rock stability evaluation.

## Figures and Tables

**Table 1:** Controlling parameters of cyclic wetting and drying test.

Treatments	Contents	Particulars
Wetting	Test site	Geotechnical laboratory
	Soak solution	Distilled water
	Temperature	Room temperature
	Duration	72 h
Drying	Test site	Geotechnical laboratory
	Heating mode	Electric dry oven
	Temperature	110 °C
	Duration	72 h

**Table 2:** Controlling parameters of Mechanics Testing System.

Aspects	Contents	Particulars
Operating conditions	Uniaxial compression	−40–60 °C
Loading parameters	Max. axial load	4600 kN
	Max. pore water pressure	70 MPa
	Servo valve sensitivity	290 HZ
	Data acquisition channels	10
	Min. sampling time	50 μs
Sample requirements	Uniaxial compression	300 mm (Max. diameter)
		600 mm (Max. height)

**Table 3:** Controlling parameters of X-ray inspection system v|tome|x s 240.

Aspects	Contents	Particulars
Operating conditions	Temperature and Relative humidity (RH)	10–30 °C, 25–70 RH
X-ray parameters	Max. voltage	240 kV
	Max. power	320 W
	Min. focal length	4.5 mm
	Detail detectability	<1 μm
	Max. voxel resolution	<2 μm
	Geometric magnification (3D)	1.46–100×
Sample manipulator	Number of axes	5
	Rotation	0–360°
	Max. sample weight	10 kg
	Max. sample size	260 mm × 400 mm (diameter × height)
	Axis speed	0.01–80 mm/s

## Data Availability

Not applicable.
